# Transcriptome profiling reveals the roles of pigment mechanisms in postharvest broccoli yellowing

**DOI:** 10.1038/s41438-019-0155-1

**Published:** 2019-06-01

**Authors:** Feng Luo, Jia-Hui Cai, Xi-Man Kong, Qian Zhou, Xin Zhou, Ying-Bo Zhao, Shu-Juan Ji

**Affiliations:** Department of Food Science, Shenyang Agricultural University, 110866 Shenyang, PR China

**Keywords:** Plant molecular biology, Transcriptomics

## Abstract

Postharvest broccoli is prone to yellowing during storage, which is the key factor leading to a reduction in value. To explore appropriate control methods, it is important to understand the mechanisms of yellowing. We analyzed the genes related to the metabolism of chlorophyll, carotenoids, and flavonoids and the transcription factors (TFs) involved in broccoli yellowing using transcriptome sequencing profiling. Broccoli stored at 10 °C showed slight yellowing on postharvest day 5 and serious symptoms on day 12. There were significant changes in chlorophyll fluorescence kinetics, mainly manifesting as a decrease in the Fv/Fm value and an increase in nonphotochemical quenching, during the yellowing process. Transcriptome sequencing profiles from samples of fresh broccoli and broccoli with slight and severe yellowing revealed 6, 5, and 4 differentially expressed genes involved in chlorophyll metabolism, carotenoid biosynthesis, and flavonoid biosynthesis, respectively. The transcription factor gene ontology categories showed that the MYB, bHLH, and bZip gene families were involved in chlorophyll metabolism. In addition, the transcription factor families included NACs and ethylene response factors (ERFs) that regulated carotenoid biosynthesis. Reverse transcription polymerase chain reaction further confirmed that bHLH66, PIF4, LOB13, NAC92, and APL were vital transcription factors that potentially regulated the *CAO* and *HYD* genes and were involved in chlorophyll metabolism and the carotenoid biosynthetic process. The flavonoid biosynthetic pathway was mainly regulated by MYBs, NACs, WRKYs, MADSs, and bZips. The results of the differentially expressed gene (DEG) and pigment content analyses indicated that the transcriptome data were accurately and positively associated with broccoli yellowing.

## Introduction

The edible part of broccoli is a green cluster of flowers that is composed of dense buds and stems. Broccoli (*Brassica oleracea* L. var. *italica*) is a vegetable with high nutritional value and notable anticancer properties^1^. However, the inflorescences and stems of postharvest broccoli are prone to wilting and yellowing, which greatly influences product quality and value. Therefore, understanding the yellowing mechanisms and their regulation is of considerable theoretical and practical importance.

Various factors can induce postharvest yellowing of fruits and vegetables, including senescence, pathogen infection (yellow leaf curl virus and chlorosis virus), free radicals, energy, and metal ions. Maldonado-Torres et al.^2^ found that Fe^2+^ and Mn^2+^ induced yellowing of Mexican lime leaves and simultaneously changed the anatomy of the leaves. Hydrangea chlorotic mottle virus (HdCMV) can lead directly to yellowing of hydrangeas. In addition, some secondary metabolites in fruits and vegetables affect the sensory color of plants. Cadrain^3^ found that the yellowing of *Arabidopsis thaliana* leaves is caused by chlorophyll degradation and anthocyanin accumulation. Thomas^4^ found that chlorophyll degradation was the major factor in the yellowing of a *Festuca pratensis* Huds. mutant. In pepper^5^ (*Capsicum* sp.), there is a correlation between capsanthin-capsorubin synthase and fruit color. Several enzymes are involved in catalyzing pigment metabolism; however, changes in plant color are usually caused by specific genes^6^. Previous studies^7^ showed that ethylene treatment significantly contributes to chlorophyll *a* degradation and yellowing, while 1-MCP treatment significantly delays the onset of yellowing. Wang et al.^8^ revealed that a modified atmosphere could inhibit the activity of respiratory enzymes and delay the yellowing of harvested broccoli. Moreover, electrostatic atomized water particle treatment^9^ and N6-benzylaminopurine treatment^10^ could inhibit chlorophyll degradation and carotenoid biosynthesis.

High-throughput RNA-seq is an effective and sensitive technology that can reveal comprehensive genic changes in plants. In recent years, transcriptome sequencing has significantly advanced molecular-level research on higher plants. In pomelo^11^ (*Citrus grandis*), cherry^12^ (*Prunus avium* L.), apple^13^, wheat^14^ (*Triticum aestivum* L.), and *Brassica napus*^15^, previous studies using transcriptome analysis have reported some key genes and transcriptome factors (TFs) involved in color change events. However, there are few reports on transcriptome analysis of broccoli, except for a study on cytokinin-treated broccoli based on proteomics and transcriptomics^16^.

In this study, high-throughput sequencing was employed to characterize the mRNA information of fresh, slightly yellowed, and severely yellowed postharvest broccoli. We conducted a pigment and chlorophyll fluorescence study of the yellowing process, and the chlorophyll metabolism and carotenoid and flavonoid biosynthetic pathways associated with broccoli yellowing were identified. Subsequently, we determined the major genes for key pathways. The upstream TFs that regulated the yellowing-related metabolic pathways were identified by analyzing the gene ontology (GO) categories. Our research aimed to explore the vital metabolic pathways in the process of broccoli yellowing and identify the key genes and their potential upstream regulatory factors.

## Results

### Changes in chlorophyll fluorescence kinetic parameters and images during broccoli yellowing

**Fig. 1:**
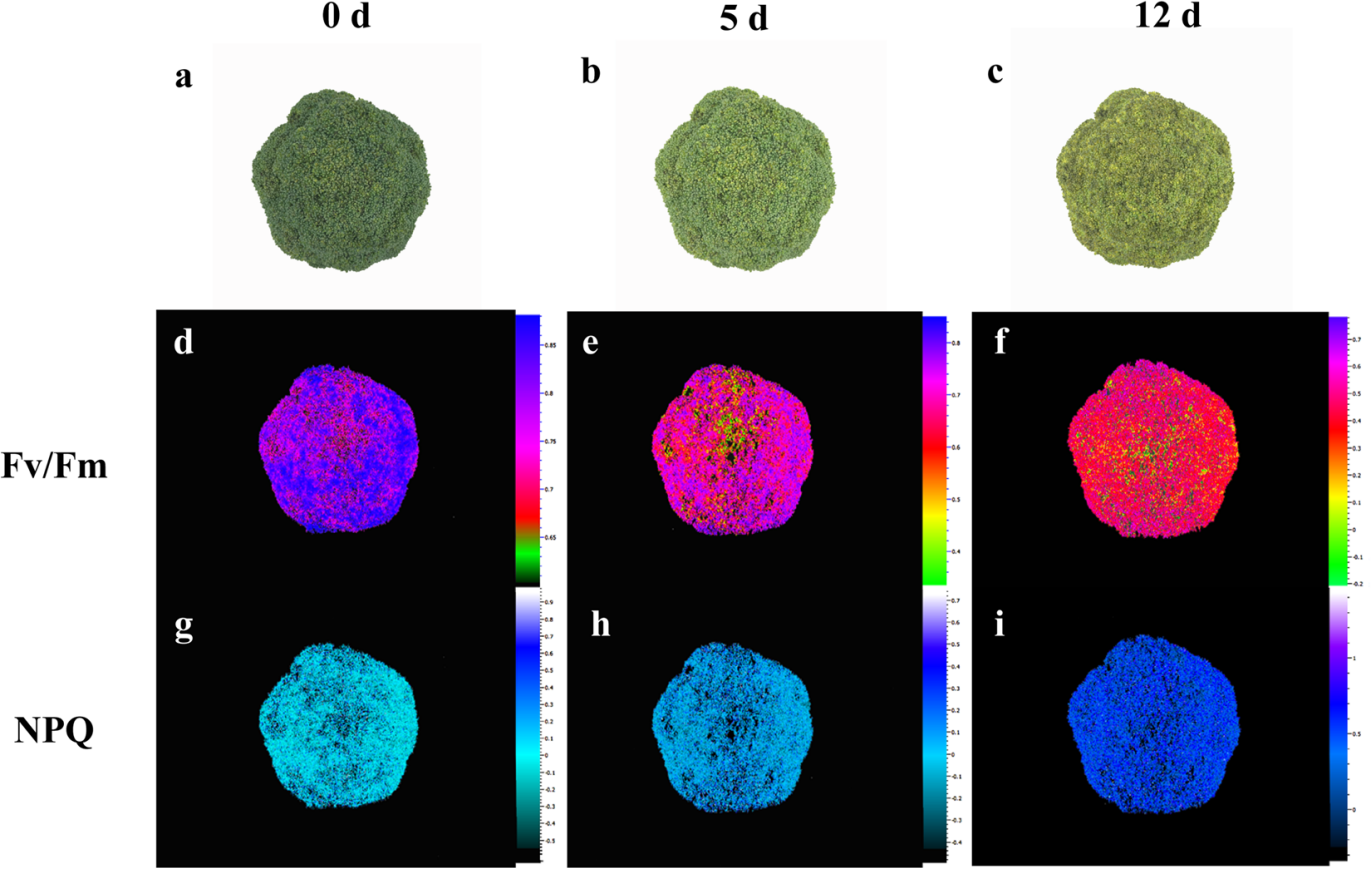
Chlorophyll fluorescence imaging of broccoli. **a−c** The sensory images obtained during broccoli yellowing. **d−i** Fluorescence images corresponding to the Fv/Fm (**d−f**) and NPQ (**g−i**) values. The images in (**d−i**) were normalized to a false color bar. Determination of the Fv/Fm ratio was carried out on dark-adapted broccoli for 15 min, while NPQ analysis was conducted at the optimum light saturation intensity

Fv/Fm is a measurement ratio that represents the maximum potential efficiency of photosystem II if all capable reaction centers are open, and nonphotochemical quenching (NPQ) is used to quantify photoprotective and photoinhibitory processes. By measuring chlorophyll fluorescence during broccoli yellowing, we obtained kinetic parameters (Table S1) and images (Fig. 1). The results showed that broccoli heads stored at 10 °C began to yellow on day 5 and showed severe yellowing on day 12 (Fig. 1a–c). During broccoli yellowing, the Fv/Fm values decreased sharply, while the NPQ values increased.

### Transcriptomic changes in broccoli during yellowing

Based on the chlorophyll fluorescence kinetic parameters, transcriptome sequencing results were obtained from three broccoli samples (fresh, slightly yellowed, and severely yellowed) with three biological replicates for each sample. A total of 68.06 Gb of clean data were obtained, and the Q30 base percentage was greater than 94.73%. Clean reads were sequentially compared with the *Brassica oleracea* genomic database, with match ratios ranging from 83.11 to 85.93%. Based on the comparison results, gene expression was analyzed using fragments per kilobase of transcript per million mapped reads (FPKM), and differentially expressed genes (DEGs) were identified according to their expression levels in different samples. As shown in Table S2, there were more DEGs in the 0 d vs. 5 d set than in the 0 d vs. 12 d set, and more DEGs appeared at 0 d than at 5 d or 12 d. The DEGs were matched and assigned to 92 KEGG pathways.

Studies on color have revealed that, in addition to DEGs, TFs play key roles in modulating color changes and the activity of pigment biosynthetic pathways. In our analysis of the transcriptome data, we focused on differentially expressed TFs. The MYB and NAC families were the most abundant TF families during the broccoli yellowing process, followed by the AP2/ERF, bHLH, WRKY, B3, and LOB families (Table 1); many of these were expressed before the samples began to yellow. Additionally, bHLHs and WRKYs were mostly upregulated before yellowing, while almost all of the abovementioned families were downregulated in the later stages of yellowing. In the preyellowing stage, 89% of B3s were downregulated, and only 2 upregulated B3s were detected when the samples yellowed. LOBs were upregulated and only detected in samples that had not yellowed. The number of AP2/ERFs was reduced by 68% after day 5.

**Table 1:** Differentially expressed transcription factors (TFs) in broccoli

DEG set	TF family	Number of DEGs	Upregulated	Downregulated	Description
0 d vs. 5 d	AP2/ERF	50	14	36	Ethylene-responsive transcription factor
	B3	27	3	24	B3 DNA-binding domain
	bHLH	46	16	30	Helix-loop-helix DNA-binding domain
	bZip	17	9	8	Basic region leucine zipper
	C2C2	34	11	23	Zinc finger protein
	C2H2	12	6	6	Zinc finger C2H2 domain-containing protein
	C3H	7	3	4	Zinc finger CCCH domain-containing protein
	HB-HD	25	9	16	Homeobox domain
	HSF	5	5	0	Heat stress transcription factor
	LOB	12	11	1	LOB domain-containing protein
	MADS	11	6	5	SRF-type transcription factor
	MYB	68	41	27	MYB-related protein
	NAC	57	47	10	NAC domain-containing protein
	WRKY	42	31	11	WRKY DNA -binding domain
	other TFs	48	30	18	
	total	461	242	219	
5 d vs. 12 d	AP2/ERF	16	6	10	Ethylene-responsive transcription factor
	B3	2	2	0	B3 DNA-binding domain
	bHLH	14	6	8	Helix-loop-helix DNA-binding domain
	bZip	4	3	1	Basic region leucine zipper
	C2C2	8	8	0	Zinc finger protein
	C2H2	1	0	1	Zinc finger C2H2 domain-containing protein
	C3H	1	1	0	Zinc finger CCCH domain-containing protein
	HB-HD	3	2	1	Homeobox domain
	HSF	3	2	1	Heat stress transcription factor
	LOB	0	0	0	LOB domain-containing protein
	MADS	1	1	0	SRF-type transcription factor
	MYB	10	6	4	MYB-related protein
	NAC	4	1	3	NAC domain-containing protein
	WRKY	21	5	16	WRKY DNA -binding domain
	other TFs	29	8	21	
	Total	117	51	66	

FDR < 0.05 and | log2 (fold change) | ≥ 1 were set as the threshold values for significantly different expression

### Chlorophyll metabolism genes involved in broccoli coloration during yellowing

**Fig. 2:**
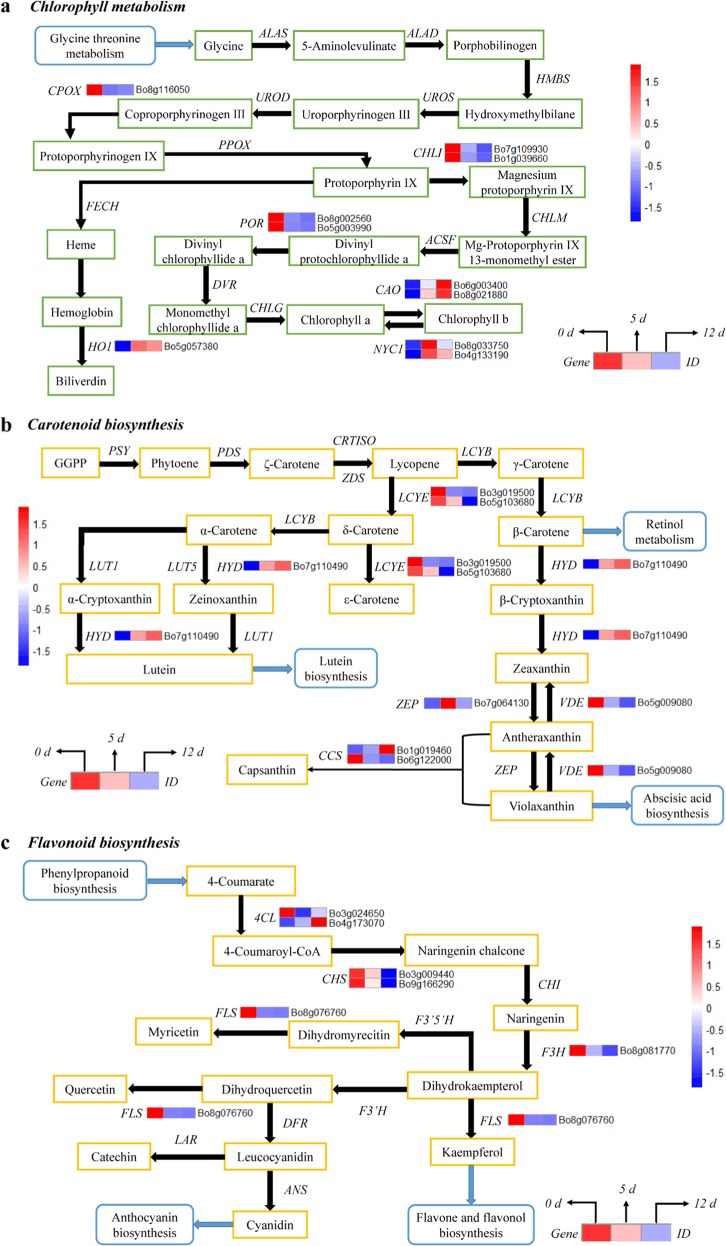
Transcriptional profiling of differentially expressed genes (DEGs) associated with pigment metabolism in broccoli during yellowing. The mean FPKM (fragments per kilobase of transcript per million mapped reads) values for the DEGs were calculated from three biological replicates for each sampling point (0, 5, and 12 d post harvest). The progression of the color scale from blue to red represents an increase in the FPKM values. **a** The DEGs involved in chlorophyll metabolism. *ALAS* 5-aminolevulinate synthase, *ALAD* porphobilinogen synthase, *HMBS* hydroxymethylbilane synthase, *UROS* uroporphyrinogen-III synthase; *UROD* uroporphyrinogen decarboxylase, *CHLI* magnesium chelatase subunit I, *HO1* heme oxygenase 1, *CHLM* magnesium-protoporphyrin O-methyltransferase, *CAO* chlorophyllide *a* oxygenase; *HEMF* coproporphyrinogen III oxidase, *DVR* divinyl chlorophyllide *a* 8-vinyl-reductase, *FECH* protoporphyrin/coproporphyrin ferrochelatase, *PPOX* protoporphyrinogen/coproporphyrinogen III oxidase, *NYC1* chlorophyll(ide) *b* reductase, *ACSF* magnesium-protoporphyrin IX monomethyl ester (oxidative) cyclase, *POR* protochlorophyllide reductase, *CHLG* chlorophyll/bacteriochlorophyll *a* synthase. **b** The DEGs involved in carotenoid biosynthesis. *PSY* phytoene synthase, *ZDS* ζ-carotene desaturase, *LCYB* lycopene β-cyclase, *LCYE* lycopene ε-cyclase, *HYD* β-carotene 3-hydroxylase, *ZEP* zeaxanthin epoxidase, *VDE* violaxanthin de-epoxidase, *LUT1* carotene epsilon-monooxygenase, *LUT5* beta-ring hydroxylase, *PDS* 15-cis-phytoene desaturase, *CRTISO* prolycopene isomerase, *CCS* capsanthin/capsorubin synthase. **c** The DEGs involved in flavonoid biosynthesis. *4CL* 4-coumarate CoA ligase, *CHS* chalcone synthase, *CHI* chalcone isomerase, *F3H* flavanone 3-hydroxylase, *F3*′*5*′*H* flavonoid 3′,5′-hydroxylase, *LAR* leucocyanidin reductase, *F3*′*H* flavonoid 3′-hydroxylase, *FLS* flavonol synthase, *DFR* dihydroflavonol 4-reductase, *ANS* anthocyanidin synthase

Six DEGs, which are the key candidate genes encoding enzymes, were associated with chlorophyll metabolism (Fig. 2a). In the process of broccoli yellowing, the expression of *CHLI* (Bo7g109930 and Bo1g039660) and *POR* (Bo8g002560 and Bo5g003990) genes was continuously downregulated, while that of two *CAO* genes (Bo6g003400 and Bo8g021880) was significantly upregulated. We also found that the expression of two *NYC1* (Bo4g133190 and Bo8g033750) genes and the *HO1* (Bo5g057380) gene was significantly upregulated in early yellowing but slightly downregulated in the later stages of yellowing. The change in the expression of the *CPOX* (Bo8g116050) gene showed exactly the opposite pattern to that of *NYC1* and *HO1*.

### Carotenoid biosynthesis genes involved in broccoli coloration during yellowing

As shown in Fig. 2b, five DEGs were annotated as key genes encoding enzymes related to carotenoid biosynthesis. The DEGs encoding *HYD* (Bo7g110490) were differentially expressed in multiple branches of this KEGG pathway and were upregulated during broccoli yellowing. Additionally, the DEGs encoding ZEP were significantly upregulated before broccoli yellowed but were then downregulated. During the process of broccoli yellowing, *LCYE* (Bo3g019500 and Bo5g103680), *VDE* (Bo5g009080), and *CCS* (Bo6g122000) were significantly downregulated, but *CCS* (Bo1g019460) was not.

### Flavonoid biosynthesis genes involved in broccoli coloration during yellowing

As shown in Fig. 2c, four DEGs were annotated in the flavonoid biosynthesis pathway; however, most secondary metabolic pathways were weakened by the downregulation of genes, including *CHS* (Bo3g009440 and Bo9g166290), *FLS* (Bo8g076760), and *F3H* (Bo8g081770), during broccoli yellowing. We found that *4CL* (Bo4g173070) was the only gene that was continuously upregulated during the yellowing process. However, another *4CL* gene (Bo3g024650) was significantly downregulated during the early stage of yellowing but was upregulated after day 5.

### TFs involved in broccoli coloration

As shown in Table 2, the TF families involved in chlorophyll metabolism, carotenoid biosynthesis, and flavonoid biosynthesis were relatively fixed and were involved in the biological process regardless of the stage of broccoli yellowing. MYBs, bHLHs, and bZips were the candidate TFs involved in regulating the chlorophyll metabolism-related pathway. NACs, bHLHs, bZips, MYBs, and ERFs have the potential to participate in the regulation of the carotenoid biosynthetic pathway. Moreover, six potential TF families involved in the regulation of flavonoid biosynthetic pathways were identified, including the MYB, NAC, WRKY, MADS, and bZip families.

**Table 2:** Details of yellowing-related gene ontology (GO) categories

Set	Pathway	Name	Accession	Ontology	TFs
0 d vs. 5 d	Chlorophyll metabolism	Chlorophyll biosynthetic process	GO:0015995	Biological process	ATHB16, ATHB5, PHL6, C3H20, ATHB12, ATHB21, ATHB7
		Chlorophyll catabolic process	GO:0015996		bHLH66, PIF4, PIF5, LOB13, NAC92, APL
		Regulation of chlorophyll biosynthetic process	GO:0010380		bHLH66, PIF4, APL, KAN2, LOB13, ARR18, ARR1, GATA6, NAC92, ARR11
	Carotenoid biosynthetic	Carotenoid biosynthetic process	GO:0016117		bHLH66, PIF4, CRABS CLAW, LOB13, SCREAM2, NAC92, ATHB9, ERF112, APL
		Positive regulation of carotenoid biosynthetic process	GO:1904143		ARF18, OFP15, ERF16, ARF11, NAC41, NAC83
		Negative regulation of carotenoid biosynthetic process	GO:1904142		PHL13, APL, ERF5, TINY, WRKY48, MYB4, ERF112
	Flavonoid biosynthetic	Flavonoid biosynthetic process	GO:0009813		Y-subunit A-4, MYB62, MYB108, MYB308
		Positive regulation of flavonoid biosynthetic process	GO:0009963		NAC83, MYC2
		Anthocyanin-containing compound biosynthetic process	GO:0009718		JUNGBRUNNEN 1, WRKY28, WRKY57, bHLH70
		Regulation of anthocyanin biosynthetic process	GO:0031540		CPC, MYB117, MADS8,MYB114
		Anthocyanin accumulation in tissues in response to UV light	GO:0043481		HDG1, HDG5, AGL9, SOC1
		Regulation of anthocyanin metabolic process	GO:0031537		ARR11, APL
5 d vs. 12 d	Chlorophyll metabolism	Chlorophyll biosynthetic process	GO:0015995	Biological process	ATHB122, ATHB20, HAT5
		Chlorophyll catabolic process	GO:0015996		bHLH66, PIF4, PIF5, NAC92, HFRI, LOB13, APL
		Regulation of chlorophyll biosynthetic process	GO:0010380		bHLH66, PIF4, GLK1, GATA21, ARR20, APL, ARR1, LOB13, NAC92
	Carotenoid biosynthetic	Carotenoid biosynthetic process	GO:0016117		bHLH66, NAC92, PIF4, ERF112, LOB13, APL
		Positive regulation of carotenoid biosynthetic process	GO:1904143		ARF18, TEM1, ARF11, NAC76, RAV1
		Negative regulation of carotenoid biosynthetic process	GO:1904142		TAG2, RAX2, APL, WRKY58, HAT5, ERF105
	Flavonoid biosynthetic	Flavonoid biosynthetic process	GO:0009813		MYB108, RAX2, MYB306, MYB308
		Positive regulation of flavonoid biosynthetic process	GO:0009963		ARR9, bHLH28, TEM1, RAV1, WRKY8, JUNGBRUNNEN 1
		Anthocyanin-containing compound biosynthetic process	GO:0009718		WRKY8, JUNGBRUNNEN 1, WRKY28, bHLH41, bHLH28
		Regulation of anthocyanin biosynthetic process	GO:0031540		MYB114
		Anthocyanin accumulation in tissues in response to UV light	GO:0043481		AGL9
		Regulation of anthocyanin metabolic process	GO:0031537		GLK1, ARR9, APRR1, ARR1, APL

### Pigment changes in broccoli

The yellowing process of broccoli was accompanied by the evolution of pigment content. As shown in Table 3, the chlorophyll *a* content gradually decreased, and the chlorophyll *b* content fluctuated. However, the contents of the four carotenoid components gradually increased, especially after day 5. The contents of zeaxanthin, β-carotene, lutein, and β-cryptoxanthin increased by 71.7%, 178.5%, 63.4%, and 703.7%, respectively, between 5 d and 12 d. The flavonoid content changed primarily at the stage of slight yellowing, decreasing by 26.7%; subsequently, there was no significant change.

**Table 3:** Evolution of pigment contents in broccoli during yellowing

Pigment content	Storage time (d)		
	0 d	5 d	12 d
Chlorophyll content			
Chlorophyll *a* (mg/kg)	1.12 ± 0.31a	0.77 ± 0.26b	0.50 ± 0.21c
Chlorophyll *b* (mg/kg)	0.23 ± 0.11b	0.38 ± 0.10a	0.18 ± 0.06c
Carotenoid content			
Zeaxanthin (mg/kg)	1.08 ± 0.05c	2.12 ± 0.81b	3.64 ± 1.01a
β-Carotene (mg/kg)	3.81 ± 1.14c	7.24 ± 2.26b	20.16 ± 6.02a
Lutein (mg/kg)	5.62 ± 1.02b	6.12 ± 2.11b	10.01 ± 2.54a
β-Cryptoxanthin (μg/kg)	14.21 ± 2.12c	26.18 ± 5.17b	210.4 ± 16.20a
Flavonoid content			
Flavonoids (μg/kg)	15.72 ± 2.62a	12.41 ± 2.07b	13.66 ± 3.28b

The values represent the means ± standard deviations of three experiments, each with three biological replicates (*n* = 9). The different letters within the rows indicate significant differences among different storage times (*P* < 0.05)

### Validation of DEG expression in the transcriptome data

**Fig. 3:**
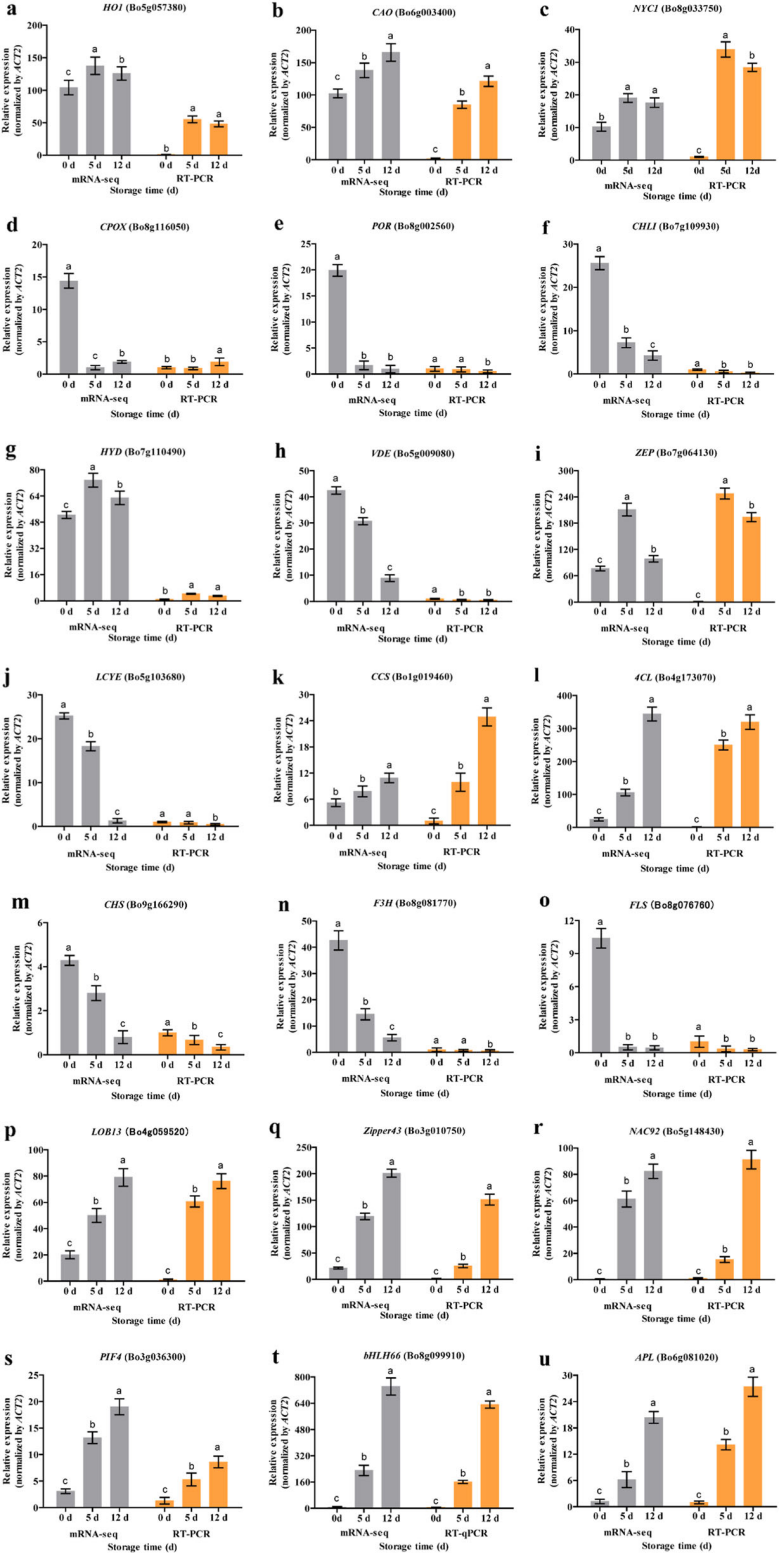
Comparison of mRNA-seq and RT-PCR analyses for 21 differentially expressed genes (DEGs) during broccoli yellowing. **a−u** DEGs associated with chlorophyll metabolism (**a−f**), carotenoid biosynthesis (**g−k**), or flavonoid biosynthesis (**l−o**) and TFs (**p−u**). The relative expression levels are plotted as FPKM values. The different letters indicate significant differences among the different sampling points (*P* < 0.05), and the error bars indicate the standard deviations

To validate the key DEG results (Fig. 3), the 21 DEGs that showed the most significant differences in expression were selected: six chlorophyll metabolism pathway genes (Fig. 3a−f), five carotenoid biosynthetic pathway genes (Fig. 3g−k), four flavonoid biosynthetic pathway genes (Fig. 3l−o), and six TFs (Fig. 3p−u). We analyzed the evolution of expression during broccoli yellowing using RT-PCR. The mRNA-seq and RT-PCR data were very closely correlated, and there was high consistency in the up- and downregulated expression of DEGs.

## Discussion

### Evolution of pigments during broccoli yellowing

Many factors are involved in postharvest broccoli yellowing. In the final analysis, the evolution of the content and the proportions of pigments were concordant. Considering these results, we suggest that early yellowing can be attributed to the accumulation of chlorophyll *b* and the lack of chlorophyll *a*, which leads to broccoli continuing to yellow after day 5. The Fv/Fm values decreased with broccoli yellowing, which reflected the gradual destruction of photosystem II (PSII) in the plant. The chloroplast thylakoid membrane is the main site of PSII, and its structure may be destroyed during the yellowing process, which would lead to a further decrease in the content of chlorophyll components^17^. Briefly, changes in chlorophyll components contributed to broccoli color variation at the pigment level.

The contents of the main carotenoid components in broccoli—β-carotenoid, lutein, and β-cryptoxanthin—continued to increase throughout the yellowing process. In particular, significant increases in the β-carotenoid and β-cryptoxanthin content were detected after day 5. Chlorophyll deficiency and carotenoid accumulation were the main reasons for broccoli yellowing. The observed increase in NPQ during the yellowing process might be due to the limitation of the photosynthetic substrate after harvest. The light energy used by broccoli is reduced, and a large part of the absorbed light quantum is dissipated in the form of heat energy and is not used to drive photosynthesis^18^. The dissipation of excess energy protects the photosystem, allowing the original electron acceptors of PSII to maintain a high oxidation state, thereby preventing damage to broccoli by the optical system. The xanthophyll cycle is one of the most important ways to release excess energy, mainly through the two-stage decyclization of violaxanthin, which was another reason for the increase in zeaxanthin content^19^.

Flavonoids have been widely studied as important yellow pigments in many fruits and vegetables; the results of these studies revealed that flavonoids are the main contributors to the yellow color of ginkgo^20^ and that they play a key role in the color of *Arabidopsis thaliana*^21^ and sweet orange during the process of fruit development and ripening. However, the decline in flavonoid levels did not contribute to the yellowing of broccoli before day 5. Although flavonoids contain flavonols, anthocyanins, and flavones, which contribute to coloration, flavonoids did not appear to be a major factor in broccoli yellowing.

### DEGs involved in chlorophyll metabolism

Chlorophyll metabolism occurs in the pigment–proteolipid complexes within thylakoid membranes that are organized to ensure a stable order of the branches in the complex chlorophyll metabolism pathway. This order is achieved by the regulation of genes involved in the pathway. Based on the transcriptome data, *CAO* was continuously upregulated throughout the process of broccoli yellowing, resulting in continuous transformation of chlorophyll *a* into chlorophyll *b* via catalysis by CAO, which resulted in chlorophyll *a* degradation. Equally significantly, chlorophyll *b* was reconverted to chlorophyll *a* via catalysis by NYC1. We found that *NYC1* was significantly upregulated before yellowing but was downregulated after day 5. The present study showed that the expression of *NYC1* and *CAO* exhibited a strong correlation with chlorophyll *b* accumulation and chlorophyll *a* degradation during broccoli yellowing. This phenomenon was the result of the ability of CAO to catalyze chlorophyll *b* synthesis, overcoming the degradation of chlorophyll *b* by NYC1. Therefore, we conclude that *CAO* plays an important role in the chlorophyll metabolism pathway during broccoli yellowing. However, this result differs from previous reports on broccoli yellowing, which concluded that *CHLI* and *PAO* were the key genes in the chlorophyll metabolism pathway^22^. Additionally, we proposed that HO1 contributed to the coloration of broccoli during yellowing. Heme oxygenase (HO) can catalyze a large amount of hemoglobin to biliverdin^23^, releasing CO and Fe^2+^, which accumulate in dark green biliverdin. HO was identified from the study of neonatal jaundice and contributes to the coloration of bird feathers and eggshells. Recently, researchers found that HO is involved in the regulation of pigment formation in plants. In *Brassica napus*^24^, defects in *HO1* could lead to a decrease in chlorophyll content. Mahawar and Shekhawat^25^ found that HO is a functionally diverse enzyme in photosynthetic organisms that plays vital roles in defense mechanisms, phytochrome chromophore biosynthesis, and cellular signaling. HO^26^ is also involved in the regulation of the anti-inflammatory activity of rhein in *Cassia fistula* L. *HO1*, as a DEG, was significantly upregulated before broccoli began to yellow and was then downregulated after day 5. This result indicates that the green pigment in broccoli is persistently synthesized during the yellowing process of broccoli and that its synthesis is gradually inhibited after yellowing begins. Collectively, these results show that chlorophyll accumulation and composition during broccoli yellowing is highly regulated by the coordinated transcriptional activation of chlorophyll metabolism genes, as has been demonstrated in some fruits.

### DEGs involved in carotenoid biosynthesis

Carotenoids—a large family of pigments that range in color from yellow to red—accumulate in large amounts in ripe fruit and senescent vegetables. However, the patterns of carotenoid biosynthesis in different species are very variable. In red-flesh navel orange, grapefruit, and pomelo, lycopene accumulation is a characteristic of carotenoid biosynthesis^27^. In our study, lutein biosynthesis, retinol metabolism, and the abscisic acid biosynthetic pathway were mainly involved in broccoli yellowing.

In addition, the key genes that influence carotenoid biosynthesis can vary among species. The expression of the 1-deoxy-d-xylulose-5-phosphate synthase (*DXS*) and hydroxymethylbutenyl diphosphate reductase (*HDR*) genes is directly related to carotenoid accumulation during tomato fruit ripening^28^. In ginkgo biloba, the upregulated genes *Z-ISO*, *ZDS*, and *LCYE* enhance carotenoid accumulation. In the present study, six DEGs were identified downstream of lycopene biosynthesis. However, PSY is the most important rate-limiting enzyme in the carotenoid biosynthesis pathway and was not differentially expressed upstream of lycopene biosynthesis during broccoli yellowing. The explanation for this observation might be that the key areas for broccoli yellowing were within and downstream of the carotenoid biosynthetic pathway.

β-Carotenoid is the most abundant carotenoid component in broccoli, and the reason for its continuous increase might be the role of *LCYB* regulation, which could offset *HYD* upregulation as a β-carotenoid downstream gene. Compared with the accumulation of other carotenoid components, the massive accumulation of β-carotenoid during yellowing appeared to correlate well with the changes in broccoli coloration. Cyclization of lycopene is a branch point: one branch leads to β-carotene; the other, α-carotene. Furthermore, the end of the α-carotene metabolic pathways points to lutein biosynthesis. The transcriptome data indicated that the only DEG in the α-carotene branch was *HYD*, and the expression of *HYD* led to noticeable accumulation of lutein after day 5. The transformation of β-carotene into β-cryptoxanthin is catalyzed by HYD, and β-cryptoxanthin is converted to zeaxanthin by the continued action of HYD. At the late yellowing stage, broccoli heads had higher proportions of β-cryptoxanthin than the previous samples. Briefly, the effect of *HYD* on carotenoid biosynthesis in broccoli was clear and played a primary role in the early stages of yellowing. The evolution of the carotenoid component was consistent with the process of postharvest broccoli coloration.

### DEGs involved in flavonoid biosynthesis

Flavonoids, as secondary metabolites of plants, are important components of plant coloration and contribute to plant resistance to external stress. Suzuki et al.^29^ found that flavonoid biosynthesis in *Torenia hybrida* could be regulated by inhibiting the expression of *CHS* and *DFR* genes, thereby changing the flower color. Similarly, Ar^30^ et al. certified that *CHS* and *DFR* genes were directly related to the coloration of petunias. In our study, four candidate DEGs associated with flavonoid biosynthesis were identified, most of which were downregulated during the process of broccoli yellowing. These genes included *CHS*, *FLS*, and *F3H*, as well as the DEGs encoding 4CL that included both downregulated and upregulated genes. The decrease in flavonoid content in the early stages of broccoli yellowing may be caused by flavonoid consumption in postharvest broccoli in response to stress. However, the flavonoid content increased slightly in the late yellowing stage of broccoli, probably due to the upregulation of *4CL* (Bo4g173070), offsetting the downregulation of *CHS*, *FLS*, and *F3H* as *4CL* downstream genes. The decreased expression of DEGs in flavonoid biosynthetic pathways and diminishing flavonoid content during postharvest storage verified that flavonoids were not directly related to the yellow coloration in broccoli.

### TFs involved in broccoli yellowing

Based on the above research on pigment and related metabolic pathway genes in broccoli, we inferred that the degradation and biosynthesis of pigments occurred throughout the entire process of yellowing in a dynamic form that was directly related to some TFs of key genes. Meng et al.^31^ found that Bell-like homeodomain 11 plays a key role in chloroplast development and chlorophyll synthesis in tomato fruit. MYC2/3/4 and NAC19/55/72 directly activate major chlorophyll catabolic genes in jasmonic acid-mediated *Arabidopsis*. Furthermore, ethylene promotes EIN3- and ORE1-mediated regulation of chlorophyll catabolic genes in *Arabidopsis*^32^. From the data for the TF GO categories, three candidate TF families involved in the chlorophyll metabolism pathway were identified: the bZip, bHLH, and MYB families. bZips are involved in the regulation of plant stress resistance^33^ and may induce the regulation of key genes during broccoli yellowing. bHLH transcription factors not only are essential for plant development but also participate in the process of plant coloration. PIF1 is a critical bHLH regulator of chlorophyll biosynthesis^34^. Studies have shown that PIF4, as a bHLH family plant pigment-interacting protein factor, could also be involved in chlorophyll metabolism^35^. MYBs are widely involved in plant development, metabolic regulation, and plant hormone signal transduction^36^, and complex metabolic activities continue to occur in broccoli after harvest.

Previous studies have shown that ERFs, NACs, EINs, and MYBs participate in the carotenoid biosynthetic process and directly regulate carotenoid accumulation. During papaya fruit ripening, EIN3a and NAC2^37^ synergistically regulate carotenoid accumulation and NAC1 activates the *PDS2/4* gene to modulate carotenoid biosynthesis. ERF6 plays an important role in carotenoid accumulation during tomato fruit ripening^38^. In kiwi fruit, MYB7 regulates chlorophyll and carotenoid accumulation^39^. In our study, the NAC, bHLH, bZip, MYB, and ERF families were the major TF families involved in the regulation of the broccoli carotenoid biosynthetic process. NACs and ERFs are the upstream TFs that regulate the ethylene signaling pathway^40^, which promotes senescence in climacteric plants (e.g., broccoli) by regulating ethylene synthesis, leading to carotenoid accumulation. Moreover, the multihormone pathway is actively initiated by NACs; the ABA signal transduction pathway starts at a branch of the carotenoid biosynthetic network^41^. In the 0 d vs. 5 d set, we found that NACs were upregulated, while over 70% of AP2/ERFs were downregulated. We hypothesized that NACs are the positive regulators of key genes in carotenoid biosynthesis in the early stage of yellowing, whereas AP2/ERFs are actively involved as negative regulators in the accumulation of carotenoids. These explanations suggest that NACs and ERFs are the main TF regulators of carotenoid biosynthesis. Recently, bHLH was found to regulate the biological pathways of bioactive components. Studies have shown that bHLHs and MYBs are involved in flavonoid biosynthesis^42^. We can conclude that carotenoids, which are biologically active components, may also be involved in the regulation of the bHLH and MYB families.

Based on the results from the TF GO categories, we found that bHLH66, PIF4, LOB13, NAC92, and APL were simultaneously enriched in the categories of chlorophyll biosynthesis, regulation of chlorophyll biosynthesis, and carotenoid biosynthetic process. We speculated that *CAO* and *HYD* expression might be regulated or coregulated by bHLH66, PIF4, LOB13, NAC92, and APL. Our results showed that the expression levels of *CAO*, *HYD*, *LOB13*, *bHLH66*, *PIF4*, *NAC92*, and *APL* genes were positively correlated. These TFs might have a positive regulatory relationship with the *CAO* and *HYD* genes; this is an area with potential for further research.

Although the flavonoids in this study were not the main factors in broccoli yellowing, the flavonoid biosynthetic pathway contains many important metabolic pathways related to coloration. In our study, the TFs associated with flavonoid biosynthesis were MYBs, NACs, WRKYs, MADSs, and bZips. Numerous reports have demonstrated that the MYB and bHLH TF families participate in flavonoid biosynthesis^43^. Previous studies showed that *CHS* in apples^44^ is regulated by MYB4 and MYB5. In strawberries^45^, anthocyanin and flavonoid compound accumulation is regulated by FcMYB1. Li et al.^20^ found that MYB39 and bHLH25 are the major TFs that regulate flavonoid biosynthesis and affected leaf coloration. Moreover, in *Arabidopsis thaliana*, MYB75, MYB90, MYB113, MYB114, and bHLH played crucial roles in regulating the expression of *DFR*, *ANS*, and *UFGT*, affecting flavonoid biosynthesis^46^. WRKYs are involved in the biosynthetic pathway regulating secondary metabolism in plants. A TTG2-like WRKY TF was reportedly involved in the regulation of vacuolar transport and flavonoid biosynthesis^47^. Jaakola et al.^48^ verified that MADS regulates the accumulation of anthocyanin in bilberry fruit. Broadly, our findings provide new insight into the intricate transcriptional regulatory network of broccoli yellowing. With continuous improvement in our understanding of the regulatory network, the perspective of further research needs to be clarified. In our next study, we shift our research to focus on exogenous treatment and plant hormone signaling in the process of broccoli yellowing.

## Conclusions

The objective of this study was to reveal the dynamic process of broccoli yellowing from a transcriptomic perspective. Broccoli stored at 10 °C began to yellow on postharvest day 5, with severe yellowing by day 12. During the yellowing process, the Fv/Fm and NPQ values showed clear responses to the changes in broccoli coloration. Chlorophyll metabolism and carotenoid biosynthesis played vital roles at different stages of yellowing. The transcriptome sequencing and RT-PCR results indicated that *CAO* and *HYD* were the key genes involved in chlorophyll *a* decline and carotenoid accumulation, respectively, and that these genes were potentially regulated simultaneously by the TFs bHLH66, PIF4, LOB13, NAC92, and APL. The DEGs involved in flavonoid biosynthesis were downregulated, and no direct association between flavonoid content and broccoli coloration was found. The measurements of the chlorophyll, carotenoid, and flavonoid contents during the yellowing process confirmed the reliability of the transcriptome data.

## Materials and methods

### Plant material and treatments

“Naihan-Youxiu” broccoli was harvested by cutting at the base of the stem in Jinzhou, Liaoning, China. The bottom of the picked broccoli retained 3−4 leaves. The picking criteria were based on similar maturity and size and a lack of physical damage and pests. The harvested products were placed in plastic boxes and transported to the laboratory at 20 °C for 40 min. After precooling until the temperature of the center of the flower balls reached 0 °C, the samples were placed in a polyethylene bag (thickness: 0.03 mm) at 80% relative humidity in the dark. The broccoli was stored at 10 °C, which was determined to be the most suitable temperature for the study of the yellowing mechanism in our previous study, for 15 d. All data for this study were measured at three sampling points: fresh (0 d), mildly yellowed (5 d), and severely yellowed (12 d). Samples were taken from the broccoli heads and were immediately frozen in liquid nitrogen and stored at −80 °C for analysis. In addition, fresh broccoli was kept for daily sensory color and chlorophyll fluorescence detection. Each experiment was performed three times with three biological replicates each (*n* = 9).

### Measurement of chlorophyll fluorescence

The broccoli heads were placed on the console, and light was blocked with a black curtain. The samples were dark-adapted for 15 min at 20 °C, and chlorophyll fluorescence was then measured using an imaging fluorometer (Open FluorCam FC 800-O/2020; Photon System Instruments, Brno, Czech Republic). The light sources comprised actinic 1 LED panels (610–620 nm, SL3500-761/762), actinic 2 superlight LED panels (4750 K, SL3500-757/758), and an additional PAR/NDVI light panel (655/735 nm, SL3500-763). Images were captured using a CCD-695 camera under the optimum light saturation intensity for broccoli. The images (820 × 560 pixels) and the Fv/Fm and NPQ values were processed using FC800-313 software (ver. 0.21.2.0) (Photon System Instruments, Brno, Czech Republic). All images and data for each measurement were automatically repeated six times. Samples from each sampling day with similar Fv/Fm levels were screened for subsequent transcriptome sequencing.

### Measurement of the chlorophyll, carotenoid, and flavonoid contents

#### Chlorophyll extraction and determination

The methods for the extraction and determination of chlorophyll *a* and *b* from broccoli were modified based on those of Mínguez-Mosquera et al.^49^. Five grams of freshly frozen tissue from each of the three samples was ground into powder with liquid nitrogen. Next, the powder samples were homogenized with MgCO_3_ and 10 mL of acetone at 4 °C for 3 h. The solution was washed with acetone five times until the tissues turned yellowish. Acetone (10 mL) was mixed with the extraction solution, and the homogenized solution was then filtered using filter paper. Subsequently, 10 mL of NaCl solution (10%, w/v) was added to the filtrate, and the aqueous phase was removed using a separatory funnel. The separation liquid was washed with Na_2_SO_4_ (20% w/v), and 10 mL of diethyl ether was added to isolate the liquid. The concentrate was obtained by rotary evaporation of the organic phase. The concentrated solution was mixed with 2 mL of acetone and passed through a 0.22 μm nylon filter. Finally, the extract was used for HPLC-MS (high-performance liquid chromatography-mass spectrometry) analysis.

HPLC-MS analysis was performed with a liquid chromatograph mass spectrometer (LCMS-8050, Shimadzu, Kyoto, Japan). All LC-MS analyses used the same source conditions, polarity, and experimental methods, namely, ESI, positive ion-mode, and MRM-type, respectively. The detailed parameters for each analyte in the MRM experiment are shown in Table S3. The injection volume was 35 μL, and the flow rate was set to 1.0 mL/min. A stainless steel (75 × 2.1 mm) column, which was packed with 2 μm C18 Spherisorb (Shim-pack GIST Series C18; Shimadzu, Kyoto, Japan), was used at 40 °C. The mobile phase solvent system was composed of solutions A and B. The mobile phase was water:ammonium acetate (0.05 M):methanol (1:1:8 by volume), and solution B was methanol:acetone (1:1 by volume). The gradient scheme was initially 75% A and 25% B for 5 min, followed by 25% A for 8 min, isocratic for 2 min, 10% A for 8 min, and 100% B for 5 min, before a return to the initial conditions for 7 min. After column equilibration, chlorophyll *a* and *b* in the samples were separated and quantified using standard calibration curves (Table S4). The standards were obtained from Dingguo Changsheng Biotechnology Co. Ltd. (Beijing, China). Three biological replicates were used for each treatment, and the experiments were performed three times.

#### Carotenoid extraction and HPLC-MS analysis

The extraction and HPLC-MS analysis of zeaxanthin, β-carotene, lutein, and β-cryptoxanthin were performed based on the methods of Luo et al.^50^. The equipment conditions used in the HPLC-MS experiment were the same as those used for the chlorophyll content analysis. The detailed parameters for each analyte in the MRM experiment were optimized as shown in Table S3. The injection volume and flow rate were 10 mL and 0.2 mL/min, respectively. Acetonitrile:methanol (0.1 M ammonium formate):dichloromethane:water (65:20:6:9 by volume) was the mobile phase solvent system, and each run terminated at an isocratic flow rate. The carotenoid contents were calculated using a calibration curve (Table S4). The carotenoid component (zeaxanthin, β-carotene, β-cryptoxanthin, and lutein) contents were expressed as mg of the pure standard equivalent per kg of fresh weight (FW). The standards were obtained from Dingguo Changsheng Biotechnology Co. Ltd. Three biological replicates were used for each treatment, and the experiments were performed three times.

#### Determination of flavonoid content

The flavonoid content was determined as described by Luo et al.^50^. Standard solutions of quercetin at different concentrations were obtained by repeating the same procedure. The quercetin standard was obtained from Dingguo Changsheng Biotechnology Co. Ltd. The flavonoid content was calculated using a calibration curve (Table S4). The total flavonoid content was expressed as mg of quercetin equivalent/g of fresh weight (FW). Three biological replicates were used for each treatment, and the experiments were performed three times.

### RNA extraction, cDNA library preparation, and real-time quantitative RT-PCR

Buds of broccoli were frozen in liquid nitrogen. An OminiPlant RNA kit (DNase I) (CWBIO, Beijing, China) was used to extract total RNA from broccoli sprouts, and a HiFiScript cDNA Synthesis kit (CWBIO) was used for qRT-PCR to reverse transcribe the extracted total RNA. Following the UltraSYBR Mixture (Low ROX) (CWBIO) manufacturer’s instructions, qRT-PCR (QuantStudio 5; Thermo Scientific, Waltham, MA, USA) was used to analyze gene expression in the cDNA template (20 µL) reaction. Before sample loading, the cDNA concentration was determined using a spectrophotometer (NanoDrop 2000; Thermo Scientific, Waltham, MA, USA). The gene expression analysis included chlorophyll catabolism genes, carotenoid biosynthesis genes, and flavonoid biosynthesis genes that were associated with color changes. In addition, the gene expression of the relevant transcription factors (the *LOB13*, *Zipper43*, *NAC92*, *PIF4*, *bHLH66*, and *APL* genes) was determined. Primer Premier 5.0 (Premier Biosoft Inc., California, USA) was used to design the primers. The primers used for qRT-PCR are listed in Table S5. Relative gene expression values were normalized to that of the *ACT2* gene. The expression of each gene was determined using three biological replicates and calculated using the 2^−ΔΔCT^ method.

### Illumina deep sequencing and identification of differentially expressed genes (DEGs)

Three biological replicate RNA samples from fresh broccoli samples and from samples with slight and severe yellowing were prepared for transcriptome sequencing experiments. The RNA concentration was measured using the NanoDrop 2000 (Thermo Scientific). RNA integrity was assessed using an RNA Nano 6000 Assay Kit on the Agilent Bioanalyzer 2100 system (Agilent Technologies, Santa Clara, CA, USA). RNA integrity assessment was followed by library preparation for transcriptome sequencing. cDNA library construction and sequencing were performed by Biomarker Biotechnology (Beijing, China). The libraries were sequenced on an Illumina HiSeq 4000 platform (Illumina, San Diego, CA, USA). To ensure high-quality data, we removed the adapter sequences and low-quality sequence reads from the data sets (including reads containing more than 10% N and reads with a quality score of *Q* ≤ 10 for more than 50% of the entire read). The clean reads were mapped to the *Brassica oleracea* genomic database (ftp://ftp.ensemblgenomes.org/pub/plants/release-25/fasta/brassica_oleracea/) using TopHat2 (ver. 2.0.4) software with the default parameters. Quantification of the gene expression levels was estimated as FPKM. Cufflinks (ver. 2.2.1) software was used in the process of quantifying gene expression, and the counts were then normalized to the FPKM values.

DESeq (ver. 1.18.0) was used to identify genes that were differentially expressed during broccoli yellowing. A fold change (FC) value of ≥4 and a false discovery rate (FDR) of <0.001 were used as criteria for selecting DEGs. Gene function was annotated based on the GO (http://www.geneontology.org/) and Kyoto Encyclopedia of Genes and Genomes (KEGG, http://www.genome.jp/kegg/) databases. A heat map was generated using R (ver. 3.5.1) software.

### Data analysis

SPSS v.20 was used for statistical analyses (SPSS Inc., Chicago, IL, USA). Data from the three biological replicates and three parallel experiments were analyzed using standard deviation analysis. Differences between groups were evaluated by significant difference analysis (Duncan’s multiple range test). Graphs were prepared using Prism 5 software (GraphPad Inc., La Jolla, CA, USA) and Adobe Illustrator software (CC 2017; Adobe Inc., San Jose, USA).

## Supplementary information

Table S5

Table S1

Table S2

Table S3

Table S4

## Data Availability

The data generated and analyzed during the current study are available in the NCBI repository.
