# Supplementary material for: Transcriptome profiling reveals the roles of pigment mechanisms in postharvest broccoli yellowing
Source: Hortic Res. 2019 Jun 1;6:74. doi: 10.1038/s41438-019-0155-1 (PMC6544632; doi:10.1038/s41438-019-0155-1)
Supplement: Supplementary file 1 — Table S5 [file 41438_2019_155_MOESM1_ESM.docx]

**Table S5 List of primers utilized in this study**

| Target gene | Sequence(5'-3') | | |
| --- | --- | --- | --- |
|  | Forward | Reverse | Product lengh |
| *ACT2* (AF044573) | TTCAAGCTGGAGCCAAGAAGGTTC | ACGAATGGTGCGAGACAGTTAGTG | 151 |
| Chlorophyll metabolism | | | |
| *HO* (LOC106295105) | CCACAACTCACCAGCTTCCACTTC | GCCGTCGTAGCTGCAATCACC | 122 |
| *CAO* (LOC106350863) | ACCATCATGCTCCTTCACGACAAG | TCGCCAACTCTCCGCCTCTG | 111 |
| *NYC1* (LOC106341846) | ACATTGTGACGATGACGAGCACTG | TAACTCCACCGAGCCAGCTATACC | 111 |
| *CPOX* (LOC106307936) | ACTTCACGCCAGCTTACATCTTCG | AGTCATCGCACCACTTCTTGAACC | 120 |
| *CHLI* (LOC106305850) | CGGCGAGACTGACGAAGTGAAC | AGCAAGCGGTATCGAGAGCAATC | 163 |
| *POR* (LOC106307221) | TTCAGGCTGCTTACTCGCTTCTTC | CAACCATGGCATTGGCGTGAAG | 128 |
| Carotenoid biosynthesis | | | |
| *HYD* (LOC106302033) | AGAGGCTTCTCGGTCTGCTACG | GCTCGACATCACTGCGGCTATTAG | 183 |
| *ZEP* (LOC106390346) | AGACGGCGGCGGAGAGTAAG | GCTCAAGTCCTTCTCGAACACCAG | 128 |
| *CCS* (LOC106296637) | CTGGCTATCGCTGATCCTTGGC | GTTCTCCGCCGTACTTCTCATCG | 109 |
| *LCYE* (LOC106296030) | AAGGCAGCGAAAAGCAGGAA | TGACCATCCATGTAGTTTCTCCG | 169 |
| *VDE* (LOC106415200) | GATACGGCGGTGCGGTTGTG | CTCTCCACCAGAGGAGGTTCAGG | 154 |
| Flavonoid biosynthesis | | | |
| *4CL* (LOC106337156) | TAGTGGTCGTCTCTTCGCCTGATC | TTCCTCCAGTGCTCGCCGTAC | 151 |
| *CHS* (LOC106312851) | GGTACACCGTCTTCGTTGGATGAG | GTTAGCCGTGCCTATCGCCAAG | 84 |
| *F3H* (LOC106307555) | CTCGGACTCAAGCGTCACACTG | GCCTGGTGGTCAGCGTTCTTG | 197 |
| *FLS* (LOC106444537) | CGTGAAGGATACGCAAGGAGGTAC | CCTGCCTTGTGTACTCCTCGTTC | 163 |
| Transcription factor | | | |
| *LOB13* (LOC103867963) | CCGAGAAGCCGCCACAATCAC | GCAGAATGGACGCAGCGAGAG | 107 |
| *Zipper43* (LOC106325179) | CTACCACGTCCGATGAAGCAACTG | GAAGCCAAGCAACCTGCGAGAG | 150 |
| *NAC92* (LOC106294382) | AACGACAAGACCTCAAGCACATCC | ACACTCACAAGAGAACGCTCCAAC | 190 |
| *PIF4* (LOC106328266) | GTGATGACCGTTGGACCGAACC | ACCAGAGGAGCCACCTGATGATG | 141 |
| *bHLH66* (LOC106308180) | CCGCCTCCGTCCTCAGATGG | ATTCATCATTCCACCTGCCGTTCC | 186 |
| *APL* (LOC106402384) | GCAGCCGCACAAGGAGTATGG | AGCTGTTCGTGCAACCTTCTCTG | 156 |
