# Supplementary material for: Transcriptome profiling reveals the roles of pigment mechanisms in postharvest broccoli yellowing
Source: Hortic Res. 2019 Jun 1;6:74. doi: 10.1038/s41438-019-0155-1 (PMC6544632; doi:10.1038/s41438-019-0155-1)
Supplement: Supplementary file 2 — Table S1 [file 41438_2019_155_MOESM2_ESM.docx]

**Table S1 Chlorophyll fluorescence kinetic parameters of broccoli during yellowing**

| Kinetic parameters | Storage time (d) | | |
| --- | --- | --- | --- |
|  | 0 d | 5 d | 12 d |
| Fv/Fm | 0.76±0.02a | 0.69±0.01b | 0.52±0.01c |
| NPQ | 0.04±0.03c | 0.17±0.01b | 0.26±0.03a |

Note: Values represent the means ± standard deviations of three experiments, each with three biological replicates (n=9). Different letters in different rows indicate significant differences between days (P < 0.05).
