# Supplementary material for: Transcriptome profiling reveals the roles of pigment mechanisms in postharvest broccoli yellowing
Source: Hortic Res. 2019 Jun 1;6:74. doi: 10.1038/s41438-019-0155-1 (PMC6544632; doi:10.1038/s41438-019-0155-1)
Supplement: Supplementary file 3 — Table S2 [file 41438_2019_155_MOESM3_ESM.docx]

**Table S2 The number of DEGs**

| DEG set | DEG number | Up-regulated | Down-regulated |
| --- | --- | --- | --- |
| 0 d vs 5 d | 4736 | 2219 | 2517 |
| 0 d vs 12 d | 6717 | 2965 | 3752 |
| 5 d vs 12 d | 1157 | 503 | 654 |
