# Supplementary material for: Transcriptome profiling reveals the roles of pigment mechanisms in postharvest broccoli yellowing
Source: Hortic Res. 2019 Jun 1;6:74. doi: 10.1038/s41438-019-0155-1 (PMC6544632; doi:10.1038/s41438-019-0155-1)
Supplement: Supplementary file 4 — Table S3 [file 41438_2019_155_MOESM4_ESM.docx]

**Table S3 MRM transitions of the analytes**

| Analyte | Precursor (m/z) | Product (m/z) | Cone voltage (V) | Collision energy(V) |
| --- | --- | --- | --- | --- |
| chlorophyll a | 908.3 | 431.1 | 26 | 15 |
| chlorophyll b | 894.5 | 615.5 | 34 | 24 |
| zeaxanthin | 537.5 | 487.6 | 20 | 17 |
| β-carotene | 431.2 | 255.3 | 38 | 30 |
| β-cryptoxanthin | 593.4 | 505.6 | 30 | 11 |
| lutein | 512.2 | 451.3 | 36 | 10 |
