# Supplementary material for: Transcriptome profiling reveals the roles of pigment mechanisms in postharvest broccoli yellowing
Source: Hortic Res. 2019 Jun 1;6:74. doi: 10.1038/s41438-019-0155-1 (PMC6544632; doi:10.1038/s41438-019-0155-1)
Supplement: Supplementary file 5 — Table S4 [file 41438_2019_155_MOESM5_ESM.docx]

**Table S4** **The logistic equation and correlation coefficient (R) with three standard samples of pigments**

| Pigments | | Logistic equation | R | Linear range (mg·L^-1^) |
| --- | --- | --- | --- | --- |
| *Chlorophyll* | Chlorophyll a (mg/kg) | y = 1.42x **–** 0.343 | 0.994 | 0-150 |
|  | Chlorophyll b (mg/kg) | y = 0.175x + 0.116 | 0.997 | 0-150 |
| *Carotenoids* | Zeaxanthin (mg/kg) | y = 0.0162x **-** 0.0211 | 0.992 | 0-250 |
|  | β-carotene (mg/kg) | y = 68.246x **-** 62.887 | 0.998 | 0.05-100 |
|  | Lutein (mg/kg) | y = 5492.5x + 7406.5 | 0.995 | 0.05-100 |
|  | β-cryptoxanthin (μg/kg) | y = 0.0053x + 0.0087 | 0.997 | 0-250 |
| *Flavonoids* | Flavonoids (μg/kg) | y = -0.0134x + 12.188 | 0.997 | 0.025-1000 |
